# New investments in primary care in Australia

**DOI:** 10.1186/1472-6963-11-39

**Published:** 2011-02-17

**Authors:** Chris Del Mar

**Affiliations:** 1 Professor of Primary Care Research, Bond University, Australia

## Abstract

There is a crisis in primary care health workforce shortages in Australia. Its government has attempted to fix this by role-substitution (replacing medical work with nursing instead). This was not completely successful. Obstacles included entrenched social roles (leading to doctors 'checking' their nurse role-substituted work) and structures (nurses subservient to doctors) - both exacerbated by primary care doctors' ageing demographic; doctors owning their own practices; doctors feeling themselves to have primary responsibility for the care delivered; and greater attraction towards independence that may have selected doctors into primary care in the first place.

Yet there is much to be optimistic about this social experiment. It was conducted, if not ideally, at least in an environment that the Australian government has enriched with capacity for research and evaluation.

## Background

The paper by Pearce et al[1], above, clearly outlines the health worker crisis that is affecting Australia. This is true for many parts of the world, not just the Third World but many countries with developed economies as well[2].

The obvious response is for governments to create new health workers at an accelerated rate, and indeed this is happening[2]. In addition the private sector is responding to the market forces by creating new university schools of medicine, for example in Brazil and India (where there are now more private schools than public)[2].

What is interesting for Australia is that the government has attempted new interventions to fix the problem. This is the basis of its role-substituting intervention - replacing some medical responsibilities by (cheaper, and more available) nursing health workers.

## Comment

Pearce et al have undertaken a fascinating dissection of the struggles that Australia is undergoing to improve its primary care health workforce[1]. What seemed the logical step of recruiting an under-utilised resource in Australia - practice nurses - by directing funding to them, has not produced exactly what was desired. There have been some counter-intuitive effects. On the face of it, the intention was to increase the teamwork, by making nurses more equal partners in the healthcare team, legitimately getting their own fees for service together with incentive grants direct to the practice, and therefore able to see patients in their own right, rather than simply as the handmaidens of the practice.

But it seems hard to change some things. There are entrenched social structures that interfere with these good intentions: doctors more commonly own their practice than nurses, (although it is interesting to note as an aside, that as more and more practices are owned by corporations, so the owners, share-holders, could be nurses); doctors still feel themselves to have more responsibility for the care delivered in 'their' practice for any healthcare team member, and feel obliged to 'check' their co-nurses work (notwithstanding the demeaning effect this casts); the old traditional role of primary care doctors ('general practitioners', 'GPs', in Australia) and practice nurses has been more master-servant than acceptable in modern times (and it is worth remembering that the mean age of qualified GPs in Australia is worryingly >50 years old[3]); and that GPs, in the past especially, may be have been selected to work in primary care because they constitutionally are more independent, and less attracted to teamwork.

Should we be surprised that change is difficult? Since so many of these changes require substantial changes in attitude, perhaps we should not.

What can we hope for in the future? Should we complain the government that their incentives were crude and not entirely effective? Or should we provide the feedback (as Pearce et al have done so admirably) and hope for better tinkering with the system?

One thing that the Australian Government should be applauded for is the establishment of a well-resourced suite of research initiatives that is strong in health service research. There is a coordinated program of research, and an institute in the Australian Capital Territory, together with smaller investments in a number of universities around the country[4,5]. This enables a closure of the loop between the introduction of initiatives and their independent evaluation.

However it can be argued that the introduction of the initiatives in the first place could be better prepared, perhaps with the input of academics, instead of politically-derived initiatives. This would undoubtedly lead to their more formal trialling and a, therefore, more rational approach to their adoption into policy thereafter. Would politicians abdicate such responsibility to a systematic approach? Or this hope for 'evidence-based policy' simply naive?

## Conclusions

Australia should be applauded for the brave attempt to address the health workforce needs in primary care, albeit clumsily delivered and not completely successful, but also for establishing and funding the health-services research needed to fix the problem better.

## Competing interests

The author declares that they have no competing interests.

## Pre-publication history

The pre-publication history for this paper can be accessed here:

http://www.biomedcentral.com/1472-6963/11/39/prepub
